# Peroxisomal 2-Hydroxyacyl-CoA Lyase Is Involved in Endogenous Biosynthesis of Heptadecanoic Acid

**DOI:** 10.3390/molecules22101718

**Published:** 2017-10-13

**Authors:** Benjamin Jenkins, Evelyn de Schryver, Paul P. Van Veldhoven, Albert Koulman

**Affiliations:** 1 NIHR BRC Core Metabolomics and Lipidomics Laboratory, University of Cambridge, Pathology Building Level 4, Addenbrooke’s Hospital, Cambridge CB2 0QQ, UK; BJJ25@medschl.cam.ac.uk; 2 Medical Research Council Elsie Widdowson Laboratory, Fulbourn Road, Cambridge CB1 9NL, UK; 3 Laboratory of Lipid Biochemistry and Protein Interactions (LIPIT), Campus Gasthuisberg—KU Leuven, Herestraat Box 601, B-3000 Leuven, Belgium; evelyndeschryver@hotmail.com (E.d.S.); paul.vanveldhoven@med.kuleuven.be (P.P.V.V.)

**Keywords:** *Hacl1*, alpha-oxidation, pentadecanoic acid, heptadecanoic acid, C17:0, biomarker, odd chain fatty acid, peroxisomes

## Abstract

Circulating heptadecanoic acid (C17:0) is reported to be a pathology risk/prognosis biomarker and a dietary biomarker. This pathology relationship has been shown to be reliably predictive even when independent of dietary contributions, suggesting that the endogenous biosynthesis of C17:0 is related to the pathological aetiology. Little is known about C17:0 biosynthesis, which tissues contribute to the circulating levels, and how C17:0 is related to pathology. *Hacl1*+/− mice were mated to obtain *Hacl1*−/− and *Hacl1*+/+ control mice. At 14 weeks, they were anesthetized for tissue collection and fatty acid analysis. Compared to *Hacl1*+/+, C15:0 was not significantly affected in any *Hacl1*−/− tissues. However, the *Hacl1*−/− plasma and liver C17:0 levels were significantly lower: ~26% and ~22%, respectively. No significant differences were seen in the different adipose tissues. To conclude, *Hacl1* plays a significant role in the liver and plasma levels of C17:0, providing evidence it can be endogenously biosynthesized via alpha-oxidation. The strong inverse association of C17:0 with pathology raises the question whether there is a direct link between α-oxidation and these diseases. Currently, there is no clear evidence, warranting further research into the role of α-oxidation in relation to metabolic diseases.

## 1. Introduction

Odd-chain fatty acids, specifically pentadecanoic acid (C15:0) and heptadecanoic acid (C17:0), have been reported to be both biomarkers of ruminant fat intake [1] as well as inversely associated with metabolic disease risk [2,3]. However, the robustness of the diet/disease biomarker correlations are appreciably different between each fatty acid [4]; for circulating C15:0, there is a stronger correlation for dietary intake than there is for C17:0, while C15:0 has a weaker inverse correlation with disease risk. C17:0 has been shown to be a reliable and reproducible biomarker of reduced risk and the rate of pathology development.

Studies have suggested that differences between C15:0 and C17:0 may be due to biosynthesis via alpha-oxidation—a one carbon atom chain shortening process within adipocytes during differentiation [5]. This work has been done in vitro and it remains unclear if adipocytes significantly contribute to circulating C15:0 and C17:0 levels in situ.

Both 3-methyl-branched chain fatty acids and straight chain fatty acids can undergo α-oxidation [6]. For the branched fatty acids (e.g., phytanic acid), the enzymes involved include phytanoyl-CoA hydroxylase (PHYH) and 2-hydroxyacyl-CoA lyase (HACL1), both located in peroxisomes [6]. PHYH introduces a 2-hydroxy group into phytanoyl-CoA, whereas HACL1 catalyses the cleavage between the first and the second carbon atoms, generating formyl-CoA and pristanal. Recent work in HACL1-deficient mice revealed the presence of an additional enzyme with lyase activity, localized in the endoplasmic reticulum, able to cleave 2-hydroxyphytanoyl-CoA [7]. The α-oxidation process of straight chain fatty acids is slightly different: based on studies in hepatocytes, it has been determined that they are 2-hydroxylated by fatty acid 2-hydroxylase (FA2H), subsequently activated, and then cleaved by HACL1 [8]. Recently, Kitamura and colleagues [9] showed in yeast and CHO cells that an orphan enzyme known as bacterial acetolactate synthase-like can also cleave straight chain 2-hydroxyacyl-CoAs. They renamed it to HACL2, likely identical to the endoplasmic reticulum-lyase described above. 

To which extent HACL1 and HACL2 contribute to the degradation of 2-hydroxy fatty acid (2-OH-FA) and odd chain fatty acids generation in vivo is not yet clear. Likely, it might differ depending on the tissue investigated and the relative expression of these lyases. Liver has a significantly higher *Hacl1* expression when compared to all other tissues [7,8,10,11,12]. Therefore, the liver could be the dominant site of even-chain fatty acid alpha-oxidation. Based on activity measurements and the accumulation of phytanoyl-CoA metabolites in liver and plasma of phytol-treated *Hacl1*−/− mice, HACL1 is the major enzyme in 2-OH-phytanoyl-CoA removal [7]. Related to adipose tissue, studies showed that brown adipose tissue (BAT) displays an increased expression of *Hacl1* over white adipose tissue (WAT) [10,11]. Although both HACL1 and HACL2 contribute to odd chain fatty acid synthesis in CHO cells (starting from 2-hydroxyhexadecanoic acid) [9], it is thought that HACL1 is the main enzyme within in vivo straight chain fatty acid α-oxidation due to the greater understanding around this enzyme.

To study the influence of *Hacl1* on the circulating and tissue levels of C15:0 and C17:0, we utilized a *Hacl1*−/− mouse model.

## 2. Results

*Hacl1*−/− mice kept on a normal chow diet did not show abnormalities in liver or adipose tissue. In the present study, the fatty acid compositions were measured in the plasma, the liver, and four types of adipose tissue: inguinal-WAT, gonadal-WAT, retroperitoneal-WAT, and interscapular-BAT. The relative absolute quantities of C15:0 and C17:0 for each of these tissues is shown in Table 1.

It is unlikely to expect a complete C17:0 deficiency in the *Hacl1*−/− group since salvage pathways compensate for a deficiency in HACL1; for example, HACL2 and dietary C17:0 still may contribute.

When comparing other fatty acids (C12:0 to C18:2), there were no other significant differences in the *Hacl1*−/− group.

## 3. Discussion

Many epidemiological studies have shown an inverse association of circulating C17:0 with metabolic disease risks. The limited correlation of circulating C17:0 with dietary sources [4] suggests that there are endogenous mechanisms that contribute to the circulating level of C17:0. Several studies have suggested that α-oxidation is responsible for the conversion of even-chain fatty acids into odd-chain fatty acids [13,14], but there has been no study providing direct evidence for its involvement in the biosynthesis of circulating odd chain fatty acids. This is mainly because one links α-oxidation primarily to the degradation of 3-methyl-branched fatty acids [15]. Additionally, very recent data indicate the existence of two pathways for odd-chain fatty acid formation: one via peroxisomal HACL1, one via the endoplasmic reticulum-localized HACL2 [7,9].

The quantities of C15:0 in all the tissues analysed did not significantly differ between the *Hacl1*−/− and the *Hacl1*+/+ groups (see Table 1), suggesting that the peroxisomal α-oxidation does not significantly contribute to the biosynthesis of C15:0. Likely, the HACL2 contribution (although acting in CHO cells on 2-OH-hexadecanoic acid [9]) is also minor, based on published literature that shows that the dietary intake of C15:0 correlates with the circulating C15:0 levels [16]. 

However, the plasma C17:0 quantities were ~26% lower in the *Hacl1*−/− group than the *Hacl1*+/+ group (see Table 1). Comparably, the hepatic C17:0 content was ~22% lower in the *Hacl1*−/− than the *Hacl1*+/+ group. It is unlikely to expect a complete C17:0 deficiency in the *Hacl1*−/− group since salvage pathways compensate for a deficiency in HACL1; for example, HACL2 and dietary C17:0 may still contribute. No adipose tissue showed any significant differences in C17:0. This does not exclude the possibility that adipose tissue can produce C17:0. Indeed, during differentiation, 3T3-L1 start to produce odd-chain fatty acids [5]. On the other hand, compared to liver, amounts of both HACL1 and HACL2 are lower in adipose tissue [7,17,18]. Our data suggest that the role of *Hacl1* in the local WAT production of odd-chain fatty acids is insignificant.

The ratio of C15:0 to C17:0 compositions in the circulation is ~1:2 [1]; this ratio can be seen in the liver tissue and BAT, but not in any of the WATs, which further suggests that the liver tissue is the biosynthetic source of circulating C17:0. The BAT also has this ~1:2 ratio, but there was no significant difference in C17:0 between the groups, suggesting its odd chain fatty acid composition may be derived from the circulation, unlike the WAT where de novo lipogenesis affects the fatty acid composition [19]. 

When looking at the change of these odd-chain fatty acids between the *Hacl1*−/− and the *Hacl1*+/+ groups, C15:0 mirrored the change in C17:0; so, in the tissues where C17:0 decreased in the knockout the C15:0 showed a similar but less attenuated decrease and therefore not significant, and vice versa. This is to be expected because C15:0 can be produced from C17:0 via peroxisomal or mitochondrial β-oxidation, but this process is slow (significantly slower than even-chain fatty acid β-oxidation) [20], and therefore not statistically significant. The minor contribution of C15:0 via β-oxidation can explain the presence of C15:0 in people (and animals) that have an extremely low C15:0 dietary content, such as vegans (data not shown).

The results for the plasma, liver, and adipose tissues are important and are supported by published literature [21]. Adipose tissue is thought to be less of a lipid excreting tissue, but more for lipid storage with a highly regulated composition [19,22]. On the other hand, the liver’s fatty acid composition can vary drastically with relatively small changes in dietary intake. It has been shown that the fatty acid composition of the adipose tissue can remain relatively constant even when dietary interventions are imposed [19,23]. This is important because it would mean that the adipose tissue may be more informative for disease risk with respect to odd-chain fatty acids [16,24,25], since its composition does not display the same short-term fluctuations as the plasma or liver.

When collating all these results together, it can be concluded that *Hacl1* plays a significant but not essential role in the biosynthesis of C17:0 in the liver tissue, which in turn influences the circulating levels. Based on the reduction in C17:0 levels observed, the contribution of HACL2 to C17:0 synthesis is likely higher. In other tissues (e.g., adipose tissue (this study) and brain [7]), no effect on odd-chain fatty acids of HACL1 deficiency was noticed. In these tissues, odd-chain fatty acid formation may be predominantly regulated via HACL2.

We expect that C15:0 can be minimally biosynthesised through peroxisomal or mitochondrial β-oxidation of C17:0, although the dietary intake of this fatty acid will have a greater influence, especially when this is translated into large clinical epidemiology studies; proving that C15:0 is a strong biomarker of intake, as previously reported [4].

This study provides further proof of the endogenous production of C17:0 in mammals. Given that both HACL1 and HACL2 are dependent on thiamine-pyrophosphate [9], one could expect an effect of thiamine deficiency on odd-chain fatty acids, but thiamine deficiency is currently very rare in Western countries. The strong inverse association of C17:0 with diabetes and cardiovascular risk raises the question of where there is direct link between α-oxidation and these diseases. There is currently no clear evidence, highlighting the importance of further investigation into novel metabolic mechanisms and how these mechanisms relate to metabolic disease development.

## 4. Materials and Methods

Heterozygous (*Hacl1*+/−) mice from a Swiss Webster background [7] were mated to obtain *Hacl1*−/− mice. Wild-type littermates (*Hacl1*+/+) were used as controls. All animals were kept under conventional conditions in the animal housing facility of the KU Leuven (KU Leuven, Leuven, Belgium), with ad libitum access to regular chow diet and water. 

At the age of 14 weeks, mice were anesthetized with Nembutal for tissue collection (*n* = 15 *Hacl1*−/−; 8 females, 7 males, *n* = 13 control (*Hacl1*+/+); 7 females, 6 males). Blood was collected by heart puncture and plasma isolated in Mini-collect lithium heparin tubes (Greiner Bio-one). Liver, WAT (inguinal, gonadal, and retroperitoneal) and interscapular BAT were collected and snap-frozen in liquid nitrogen. All samples were stored at −80 °C until analysed (fatty acid analysis by GC-MS).

All animal experiments were approved by the Institutional Animal Ethical Committee of KU Leuven.

For the extraction of fatty acid, chloroform:methanol solution (2:1, 1 mL) and water (400 μL) were added to 50 μL of plasma or 25 mg of tissue. After tissue-lysis, samples were centrifuged (~20,000× *g*, 5 min). To the dried organic extracts, boron trifluoride in methanol (14%, 125 μL), chloroform:methanol (1:1, 100 μL), and internal standard tridecanoic acid-*_d_*_25_ in chloroform (100 μL, 200 μMol/L) were added. Samples were heated to 80 °C for 90 min. After cooling, water (300 μL) and hexane (600 μL) were added. The organic layer was dried and reconstituted in hexane (200 μL) for gas chromatography with mass spectrometry analysis. 

Gas chromatography with mass spectrometry detection was achieved using a 6890N/5973 system (Agilent Technologies, Santa Clara, CA, USA) with a HP-88 column (30 m, 0.25 mm, 0.2 μm). The front inlet temperature was 250 °C. The injection volume for plasma and liver samples was 1 μL, for adipose tissue samples the injection volume was reduced to 0.1 μL. Front inlet split ratio for plasma samples was 10:1, liver and adipose tissue samples had a 50:1 split ratio. A total oven gradient over ~28 min from 120 °C to 240 °C (initial temperature 120 °C hold of 1 min, temperature increase of 10 °C per minute to 170 °C followed by a hold of 6 min, then temperature increase of 3 °C per minute to 194 °C, then an increase of 43 °C per minute to 240 °C, followed by a hold of 7 min). Full scan detection 60–400 Da, transfer line temperature of 280 °C, source temperature of 230 °C, and quadrupole temperature of 150 °C.

## Figures and Tables

**Table 1:** The relative quantities of pentadecanoic acid (C15:0) and heptadecanoic acid (C17:0) when comparing *Hacl1*−/− mice and the related *Hacl1*+/+ control mice in each of the studied tissues: plasma, liver, three white adipose tissues (inguinal; I-WAT, gonadal; G-WAT, and retroperitoneal; R-WAT) and interscapular brown adipose tissue (I-BAT). The samples were analysed by gas chromatography with mass spectrometry detection and normalized to an internal standard to account for analytical variation, tissue absolute quantities normalized to total lipid content (nMol/g of fat). The significance of the difference between the *Hacl1* knockout mice (*Hacl1*−/−) and the related wildtype mice (control) are shown by the *t*-test; a value of *p* ≤ 0.05 was considered significant; * *p* < 0.01, ** *p* < 0.001. Results are shown with ± standard error of the mean. (*n* = 13–15 per group).

	C15:0 (nMol/g)		C17:0 (nMol/g)	
	Control	*Hacl1*−/−	Control	*Hacl1*−/−
**Plasma**	18.2 ± 1.9	16.2 ± 1.1	39.0 ± 3.1	28.7 ± 1.3 *
**Liver**	22.0 ± 1.2	20.6 ± 1.0	53.8 ± 2.4	42.2 ± 1.9 **
**I-WAT**	24.0 ± 2.0	25.0 ± 1.6	24.6 ± 1.8	26.1 ± 1.2
**G-WAT**	25.9 ± 1.2	23.6 ± 1.0	30.8 ± 1.2	28.7 ± 0.7
**R-WAT**	25.7 ± 1.1	25.5 ± 1.0	29.9 ± 1.7	30.6 ± 1.0
**I-BAT**	8.5 ± 0.4	8.0 ± 0.5	18.1 ± 1.0	16.9 ± 0.9
